# Author Correction: Visualizing group II intron dynamics between the first and second steps of splicing

**DOI:** 10.1038/s41467-021-27699-2

**Published:** 2022-01-04

**Authors:** Jacopo Manigrasso, Isabel Chillón, Vito Genna, Pietro Vidossich, Srinivas Somarowthu, Anna Marie Pyle, Marco De Vivo, Marco Marcia

**Affiliations:** 1 Laboratory of Molecular Modelling & Drug Discovery, Istituto Italiano di Tecnologia, Via Morego 30, 16163 Genoa, Italy; 2 European Molecular Biology Laboratory (EMBL) Grenoble, 71 Avenue des Martyrs, Grenoble, 38042 France; 3 Department of Structural and Computational Biology, Institute for Research in Biomedicine (IRB), Parc Científic de Barcelona, C/ Baldiri Reixac 10-12, 08028 Barcelona, Spain; 4 Department of Biochemistry & Molecular Biology, Drexel University College of Medicine, Philadelphia, PA USA; 5 Department of Molecular, Cellular and Developmental Biology, New Haven, CT 06511 USA; 6 Department of Chemistry, Yale University, New Haven, CT 06511 USA; 7 Howard Hughes Medical Institute, Chevy Chase, MD 20815 USA

**Keywords:** RNA, Structural biology, Computational biology and bioinformatics

Correction to: *Nature Communications* 10.1038/s41467-020-16741-4, published online 05 June 2020.

In the original version of the published article, several funding sources were omitted from the “Acknowledgements” section. The correct acknowledgement paragraph is:

This work is based upon research conducted at the Northeastern Collaborative Access Team beamlines, which are funded by the National Institute of General Medical Sciences from the National Institutes of Health (P30 GM124165). The Eiger 16M detector on 24-ID-E is funded by an NIH-ORIP HEI grant (S10OD021527). This research used resources of the Advanced Photon Source, a US Department of Energy (DOE) Office of Science User Facility operated for the DOE Office of Science by Argonne National Laboratory under Contract No. DE-AC02-06CH11357. We would also like to thank Dr. Laura Murray for help in cloning and crystallizing the G and U mutants, Gabriele Drews for technical assistance, and Dr. Olga Fedorova for the critical reading of the manuscript. We also thank all members of the Marcia, Pyle, and De Vivo labs for helpful discussion. Work in the Marcia lab is partly funded by the Agence Nationale de la Recherche (ANR-15-CE11-0003-01), by the Agence Nationale de Recherche sur le Sida et les hépatites virales (ANRS) (ECTZ18552), by ITMO Cancer (18CN047-00), and by the Fondation ARC pour la recherche sur le cancer (PJA20191209284). The Marcia lab uses the platforms of the Grenoble Instruct Center (ISBG: UMS 3518 CNRS-CEA-UJF-EMBL) with support from FRISBI (ANR-10-INSB-05-02) and GRAL (ANR-10-LABX-49-01) within the Grenoble Partnership for Structural Biology (PSB). M.D.V. thanks the Italian Association for Cancer Research (AIRC) for financial support (IG 23679). A.M.P. is a Howard Hughes Medical Institute investigator.

This has now been corrected in both the PDF and HTML versions of the Article.

