# Supplementary material for: Visualizing group II intron dynamics between the first and second steps of splicing
Source: Nat Commun. 2020 Jun 5;11:2837. doi: 10.1038/s41467-020-16741-4 (PMC7275048; doi:10.1038/s41467-020-16741-4)
Supplement: Supplementary file 1 — Supplementary Information [file 41467_2020_16741_MOESM1_ESM.pdf]

SUPPLEMENTARY INFORMATION FILE FOR:

Visualizing group II intron dynamics between the first and second steps of splicing

Manigrasso et al.

This Supplementary Information File includes:

- Supplementary Figures 1-11
- Supplementary Tables 1-2

Supplementary Figures

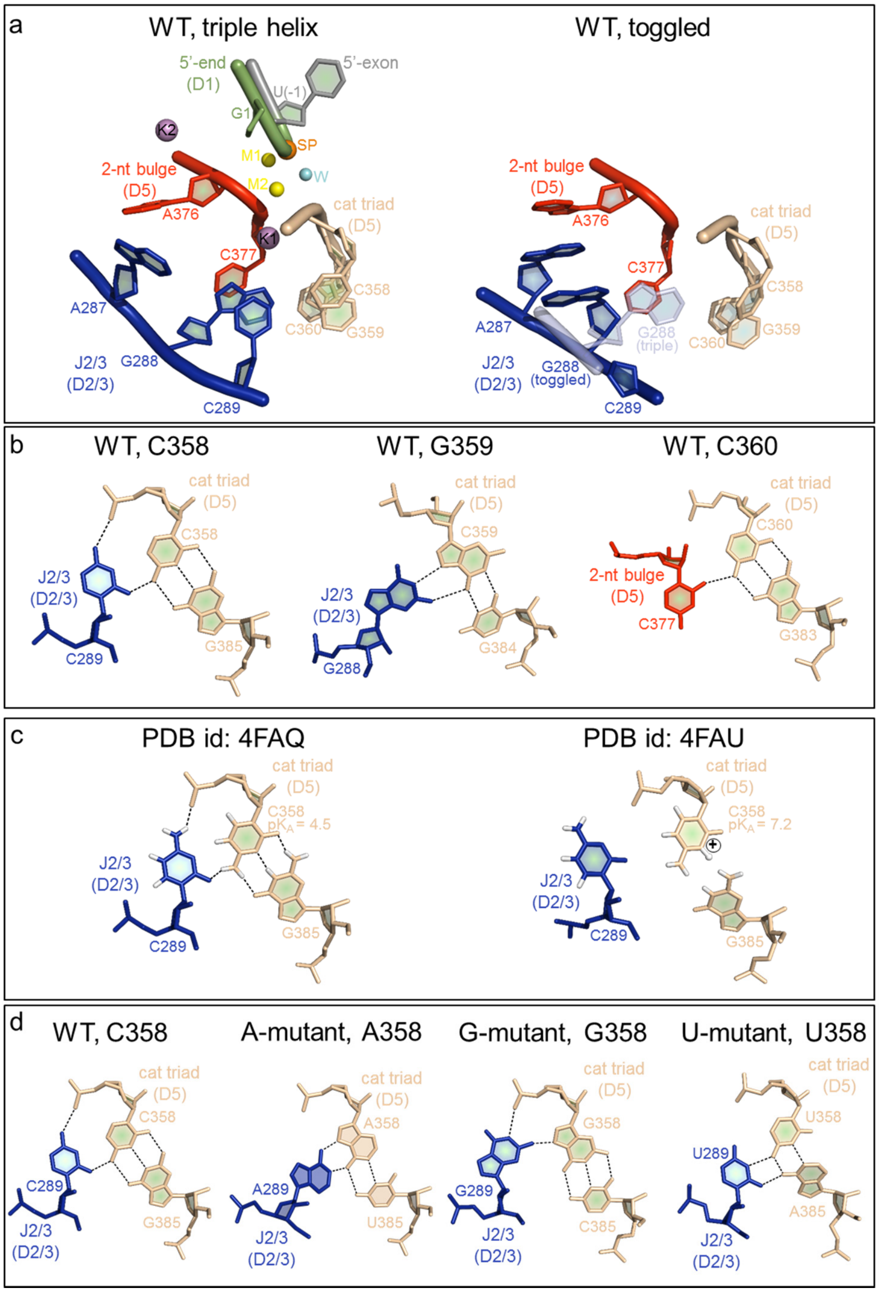

Supplementary Figure 1. Triple helix and toggled conformations in group II intron. a) The active site of the pre-hydrolytic intron (PDB id.: 4FAQ, left) and of the toggled intron (PDB id.: 4FAX, right). J2/3 junction is in blue, two-nucleotide bulge in red, catalytic triad in brown, intron 5'-end in green, and 5' exon in gray. Catalytic magnesium ions M1-M2 are depicted as yellow spheres, potassium ions K1-K2 as violet spheres, the nucleophilic water molecule is in cyan, and the scissile phosphate (SP) in orange. b) Triple interactions for the three nucleotides of the catalytic triad (from left to right: C358, G359, and C360) in wild type. c) Left: C358 in the pre-hydrolytic state forms a triple interaction, as observed in PDB id.: 4FAQ. In this state, the pK_A of C358 is 4.5, so that its nucleobase is not protonated. Right: In the toggled structure, the triple interaction is disrupted, as observed in PDB id.: 4FAX. In this state, the pK_A of C358 is 7.2, so that its nucleobase can be protonated on the N3 atom. Hydrogen bonds are depicted as black dotted lines, and hydrogen atoms are in white. d) From left to right: triple interactions of residue 358 in wild type (same as panel c, left), A-mutant, G-mutant, and U-mutant.

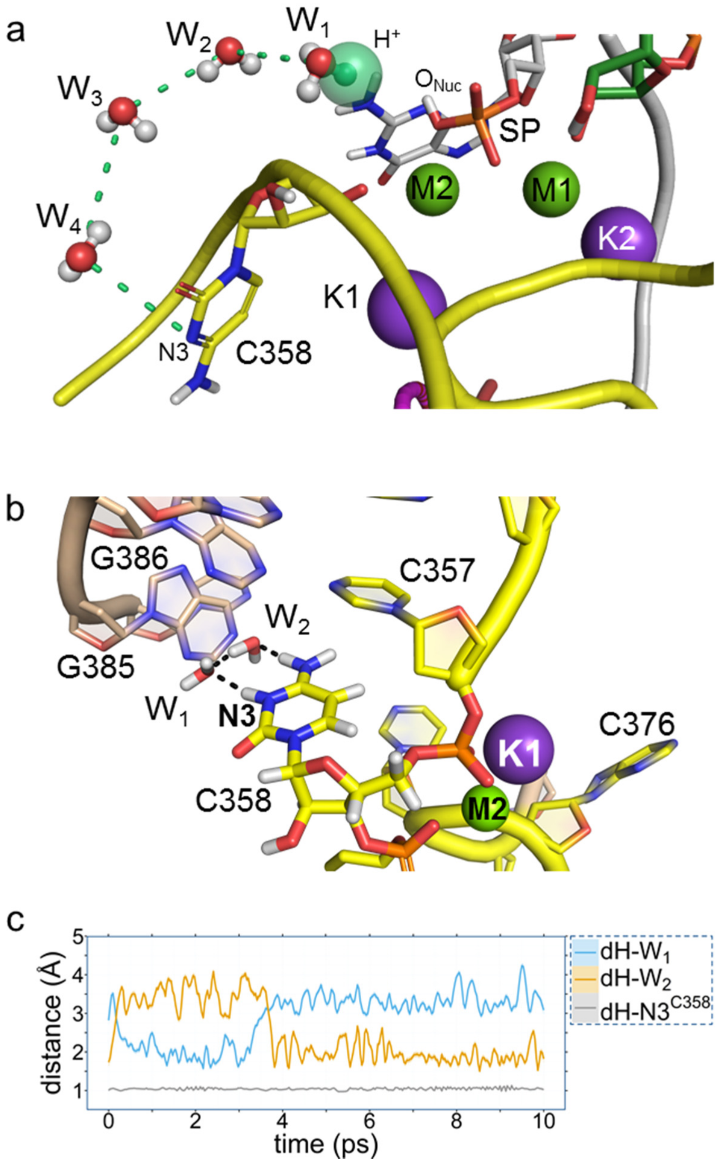

Supplementary Figure 2. N3^{C358} can be protonated after the first step of splicing. a) Active site representation of the solvated structural model of the cleaved intron. The nucleophilic water (O_{Nuc}) has attacked the scissile phosphate (SP) and released a proton (H^+, semi-transparent dark green sphere) to a neighboring water molecule in the bulk solvent (W_1). A proton shuttling pathway composed of 3 water molecules at H-bond distances from one other (W_2-W_4) can conduct this proton to N3^{C358} (green dashed lines). Other proton transfer pathways would also be compatible with our structures and can count up to 5 water molecules, based on previously reported QM/MM calculations[32]. Alternatively, proton transfer can also occur by unspecific diffusion through the bulk solvent. b) A representative snapshot of intron active site during hybrid quantum (DFT/BLYP)/classical simulations of N3^{C358} protonated in the cleaved state. Dotted lines highlight H-bonds between C358 and nearby water molecules. The intron backbone is represented as a cartoon (catalytic triad and two-nucleotide bulge in yellow, triple helix partner nucleotides in brown). M2 and K1 are represented as green and purple spheres, respectively. Atoms in bold sticks are treated at the quantum level, while the rest of the atoms are treated at the classical level. c) Hybrid quantum/classical simulations of N3^{C358} protonated in the cleaved state. Time series of selected distances during the last 10 ps of the simulations (two water molecules, in the quantum region, exchange their position relative to N3^{C358} at ~3.5 ps): $d_{H\text{-}N3C358}$ (grey trace), $d_{H\text{-}WAT2}$ (orange trace), $d_{H\text{-}WAT1}$ (light-blue trace). Notably, N3^{C358} remained protonated throughout the simulation time (~15 ps), never exchanging its proton with the nearby water molecules.

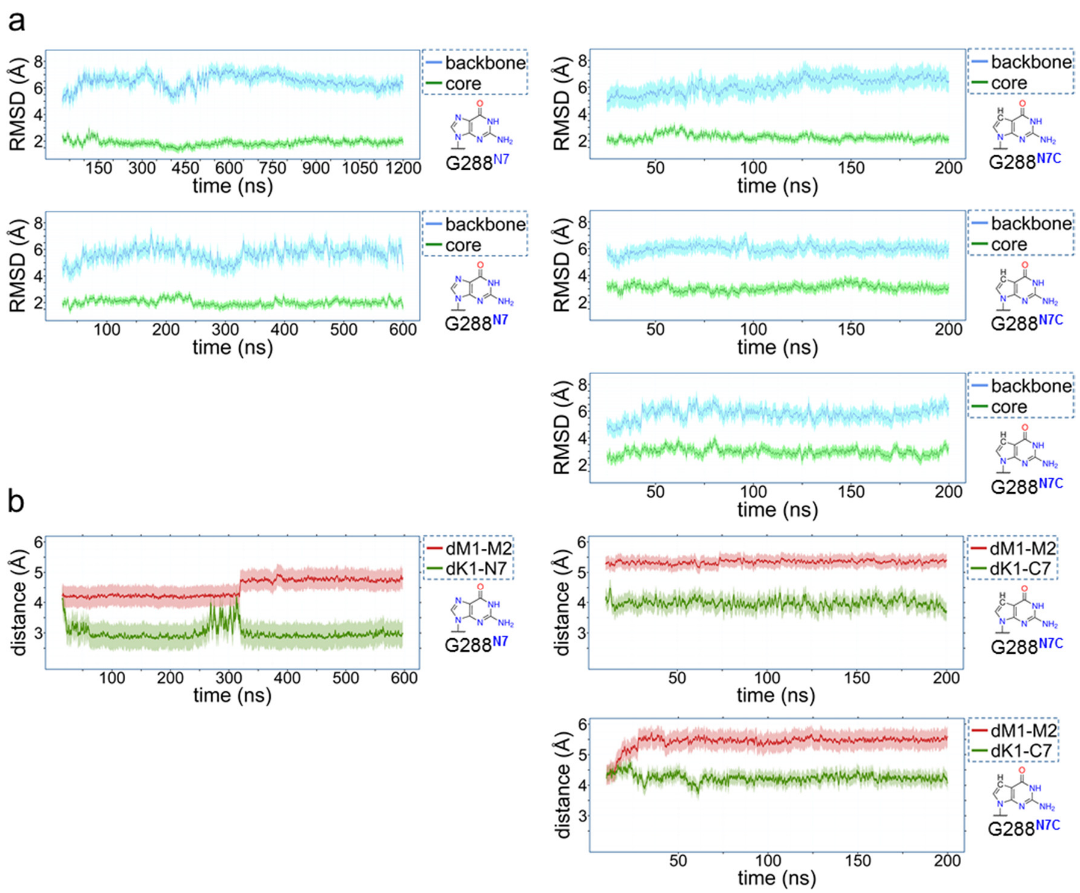

Supplementary Figure 3. MD simulations of the pre-reactive state (PDB id: 4FAQ). a) Changes in RMSD for intron backbone (blue trace) and core (defined as the backbone and side chains of all nucleotides in D5 *plus* nucleotides 287-289 of the J2/3 junction, green trace) during the two simulations of the wild type pre-reactive state (left) and during the three simulations of the $N7^{G288}$-deaza mutant pre-reactive state (right). b) Changes in $d_{K1\text{-}N7G288}$ (green trace), $d_{M1\text{-}M2}$ (red trace), during the 600-ns long MD simulation of the pre-reactive state (left; 1200-ns long simulation reported in Figure 4). In this simulation, K1 transiently drifted away from the active site after ~280 ns, causing a shift of G288 and M1-M2 ($d_{M1\text{-}M2}$ = 4.77 ± 0.16 Å). However, in contrast with the simulations of the cleaved state (Figure 5), here K1 was unable to leave the active site spontaneously. The concerted shift of K1, M1-M2, and G288 in this simulation confirms that the reciprocal structural position of these residues is intimately interconnected. On the right, changes in $d_{K1\text{-}N7G288}$ (green trace), $d_{M1\text{-}M2}$ (red trace), during two additional 200-ns-long replicas of the N7-deaza mutant pre-reactive state. Shading around the traces indicates the standard deviation of the corresponding distance.

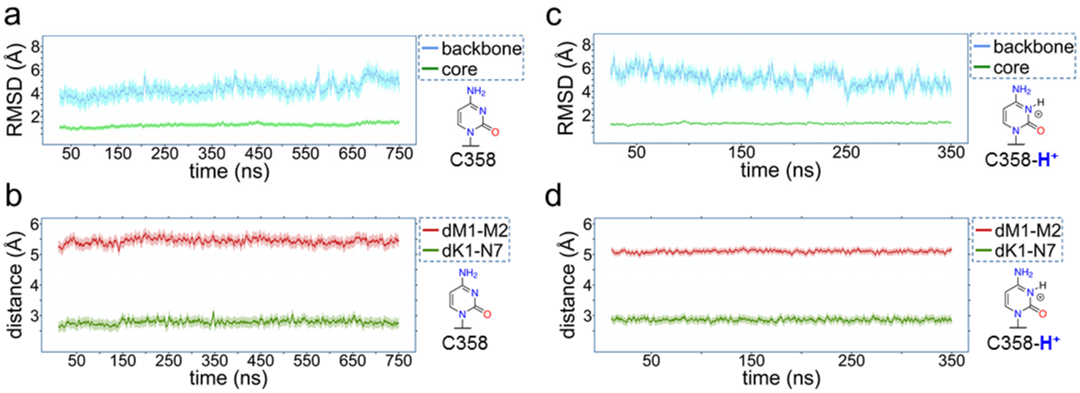

Supplementary Figure 4. MD simulations of the post-hydrolytic state (PDB id: 4FAR). a) Changes in RMSD during the simulations of the post-hydrolytic state for the backbone of all intron nucleotides (blue trace) and for the intron "core" (i.e. backbone and side chains of all nucleotides in D5 *plus* the three nucleotides of the J2/3 junction; green trace). b) Evolution of $d_{K1\text{-}N7G288}$ (green trace) and $d_{M1\text{-}M2}$ (red trace) during the simulations of the post-hydrolytic state. c) Changes in RMSD during the simulations of the post-hydrolytic H^+ state for the intron backbone (blue trace) and the intron core (green trace). d) Evolution of $d_{K1\text{-}N7G288}$ (green trace) and $d_{M1\text{-}M2}$ (red trace) during the simulations of the post-hydrolytic H^+ state. Shading around the traces indicates the standard deviation of the corresponding distance.

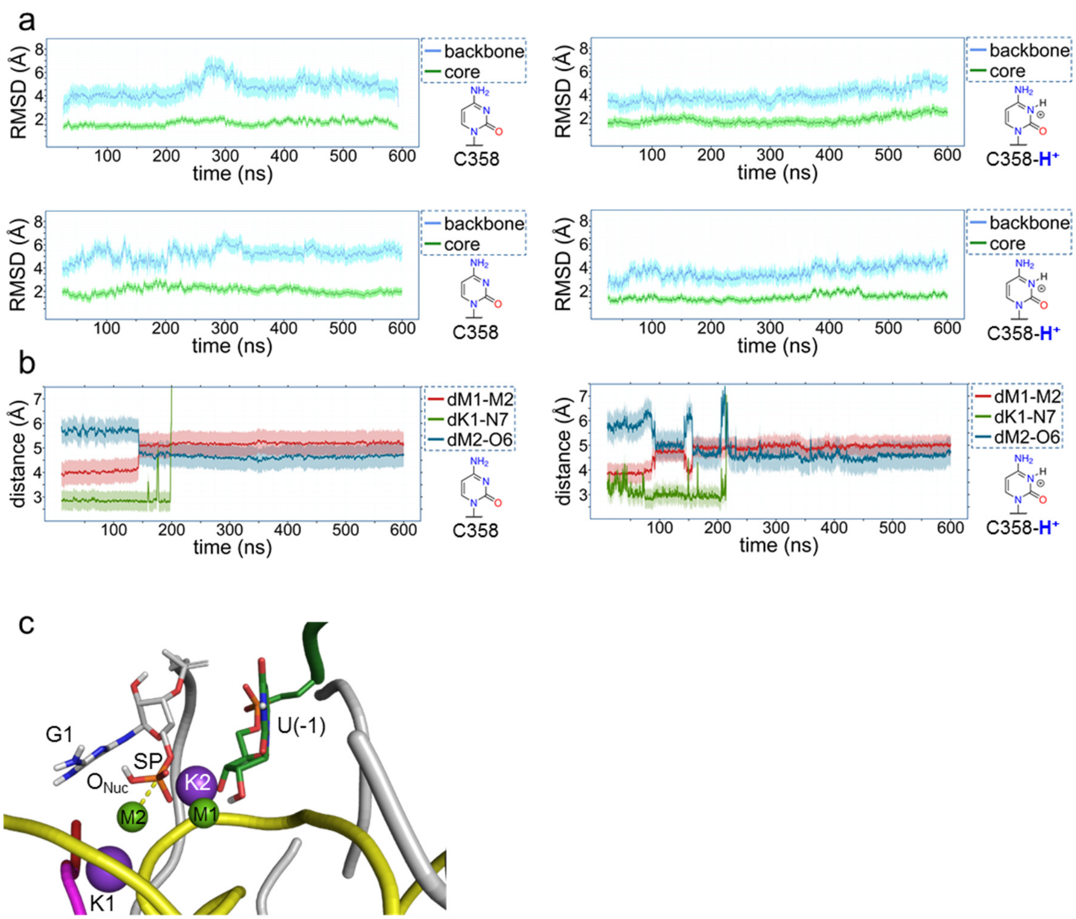

Supplementary Figure 5. MD simulations of the cleaved state of wild-type (modeled from PDB id: 4FAQ). a) Changes in RMSD during the two simulations of the cleaved state of the non-protonated (left) and protonated (right) wild type intron for the backbone (blue trace) and the core (green trace, defined as in Figure S3). b) Changes in $d_{K1\text{-}N7G288}$ (green trace), $d_{M1\text{-}M2}$ (red trace), and $d_{M2\text{-}O6}$ (blue trace) during the second MD simulation of the cleaved state of the non-protonated (left) and protonated (right) wild type intron. c) The active site of the pre-hydrolytic intron (PDB id: 4FAQ) modified to cleave the 5'-splice junction. The intron backbone is represented as a cartoon representation (catalytic triad and two-nucleotide bulge in yellow, J2/3 junction in purple, 5'-exon in green). M1 and M2 are represented as green spheres. The scissile phosphate (SP) is represented as sticks in the cleaved configuration. Oxygen atoms are red (O_{Nuc} is the oxygen atom contributed by the nucleophile), hydrogen atoms white, and the phosphorus atom is orange. The dashed yellow line indicates the distance between the scissile phosphate (SP) and M2 ($d_{SP\text{-}M2}$). Shading around the traces in panels a and b indicates the standard deviation of the corresponding distance.

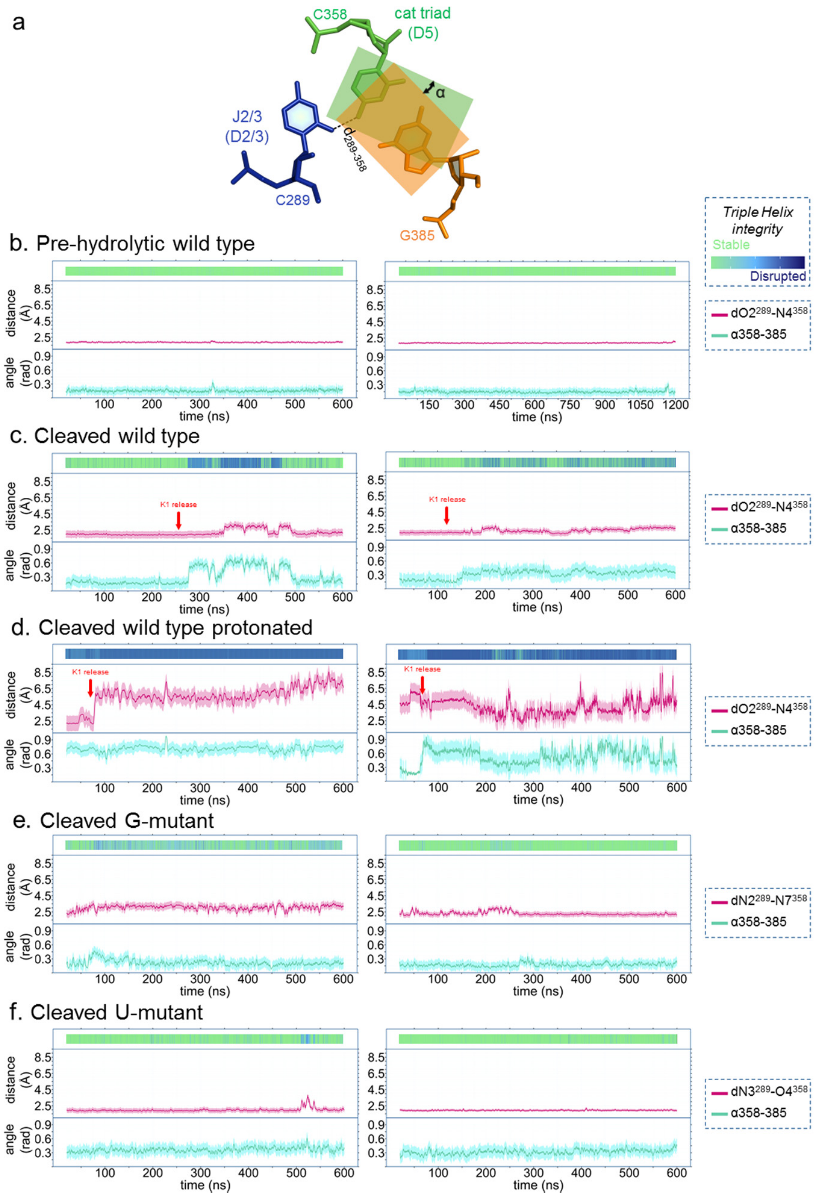

Supplementary Figure 6. K1 release induces the destabilization of the triple helix active site. a) Definition of the two parameters used to monitor the geometry of the intron triple helix at position 289: $d_{289\text{-}358}$ corresponds to the distance between C289^{O2} and C358^{N4} (black dotted line); α corresponds to the dihedral angle between the planes of the nucleobases of residues 358 and 385 (here represented as semi-transparent green and orange planes, respectively). b) Changes of $d_{289\text{-}358}$ and α during the two simulations of the pre-hydrolytic state of wild type intron. c-d) Changes of $d_{289\text{-}358}$ and α during the two simulations of the non-protonated cleaved state (c) and protonated cleaved state (d) of wild type intron. e-f) Changes of $d_{289\text{-}358}$ and α during the two simulations of the cleaved states of the G- (e) and U-mutants (f). In each plot, the colored barcode plotted on top of every MD trajectory monitors the integrity of the triple helix. When the triple helix is formed ($d_{289\text{-}358}$ < 3 Å and α < 0.35 rad) the bar is green, when the triple helix is disrupted ($d_{289\text{-}358}$ > 3 Å or α > 0.35 rad) the bar is dark blue. Release of K1 in the protonated (d) and non-protonated (c) cleaved states of wild type intron (red arrows) favors the destabilization of the triple helix. The strongest destabilization occurs in the protonated cleaved state of wild type (d). Instead, in the cleaved states of the mutants (e and f), K1 is not released into the bulk water, and the triple helix remains stable. The color scale on top of each graph in panels b-f represent a stable triple helix (green) or disrupted triple helix (blue) configuration at any given time point. Shading around the traces in panels b-f indicates the standard deviation of the corresponding distance.

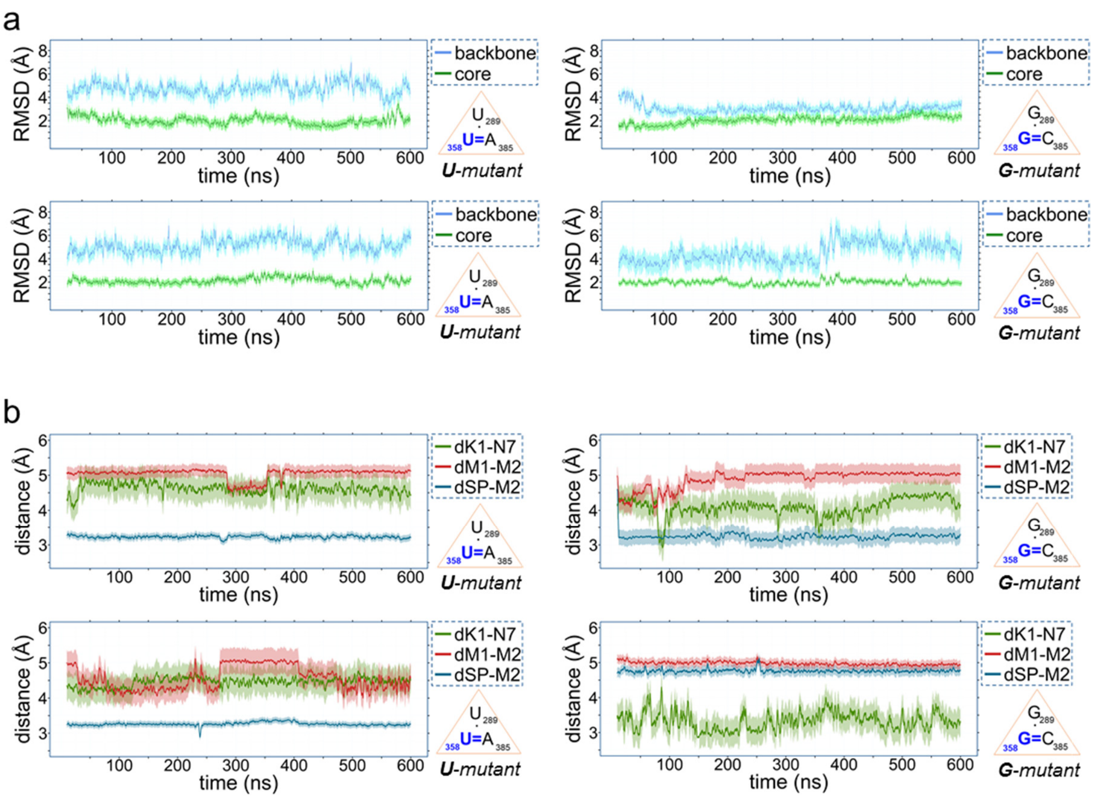

Supplementary Figure 7. MD simulations of the post-hydrolytic state of the mutants (PDB id: 6T3K and 6T3R). a) Changes in RMSD during the two simulations of the post-hydrolytic states of the U- (left) and G- (right) mutants for intron backbone (blue trace) and core (green trace, defined as in Figure S3). b) Changes in $d_{K1\text{-}N7G288}$ (green trace), $d_{M1\text{-}M2}$ (red trace), and $d_{SP\text{-}M2}$ (blue trace) during the 600-ns-long MD simulation of the post-hydrolytic states of the U- (left) and G- (right) mutants. The SP becomes coordinated by the catalytic metal cluster ($d_{SP\text{-}M2}$ = 3.63 ± 0.18 Å) while K1-N7^{G288} interaction is only transiently established and K1 is not released into the bulk water ($d_{M1\text{-}M2}$ = 4.87 ± 0.24 Å). Shading around the traces indicates the standard deviation of the corresponding distance.

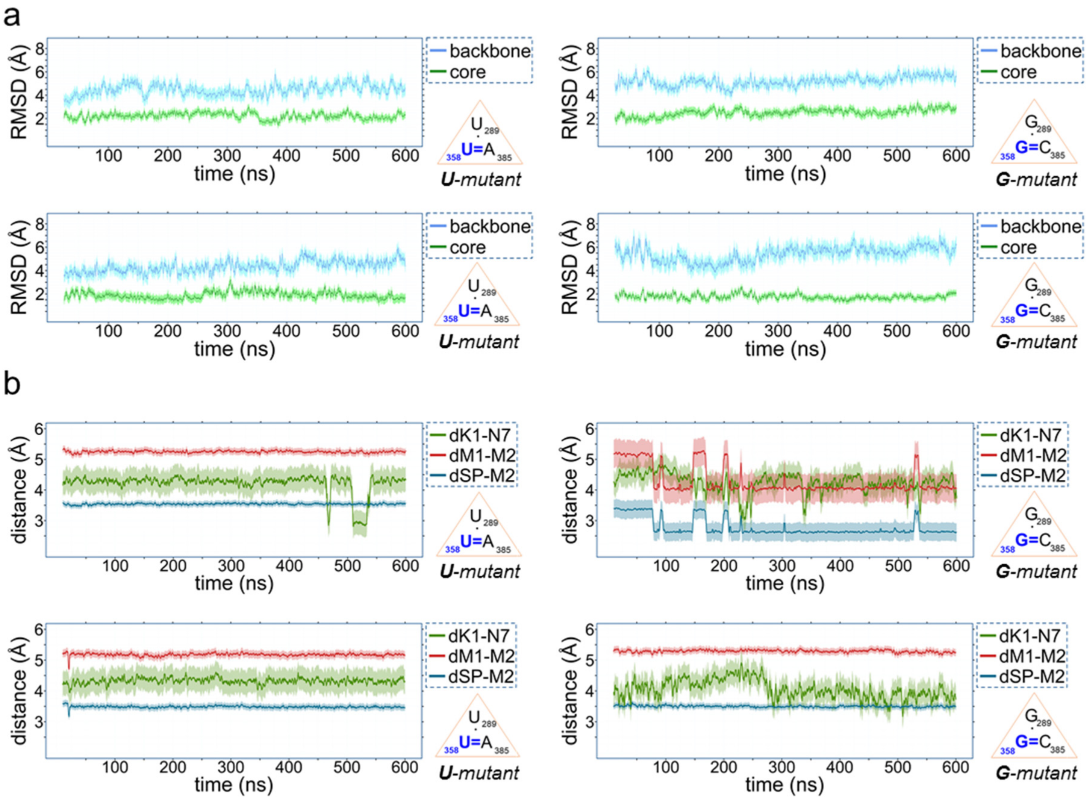

Supplementary Figure 8. MD simulations of the cleaved state of the mutants (modeled from PDB id: 6T3K and 6T3R). a) Changes in RMSD during the two simulations of the cleaved states of the U- (left) and G- (right) mutants for intron backbone (blue trace) and core (green trace, defined as in Figure S3). b) Changes in $d_{K1\text{-}N7G288}$ (green trace), $d_{M1\text{-}M2}$ (red trace), and $d_{SP\text{-}M2}$ (blue trace) during the 600-ns-long MD simulation of the cleaved states of the U- (left) and G- (right) mutants. The SP is firmly coordinated by the catalytic metal cluster ($d_{SP\text{-}M2}$ = 3.33 ± 0.15 Å) while the K1-N7^{G288} interaction is only transiently established and K1 is not released into the bulk water ($d_{M1\text{-}M2}$ = 4.21 ± 0.40 Å). Shading around the traces indicates the standard deviation of the corresponding distance.

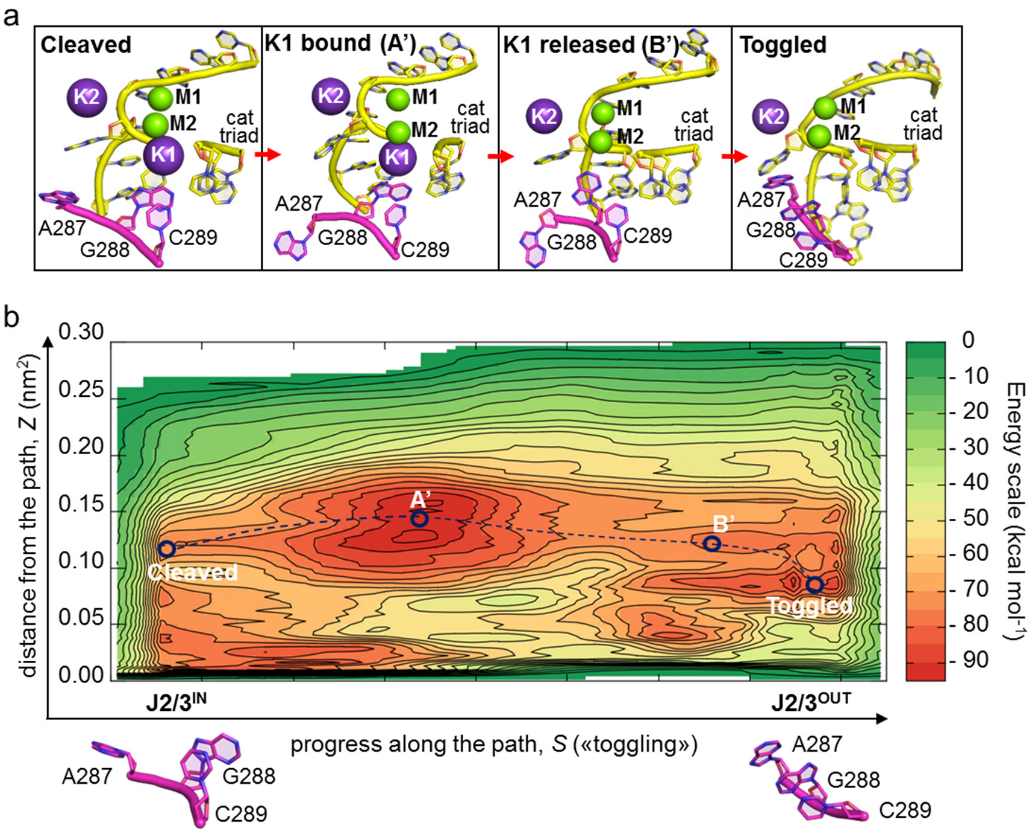

Supplementary Figure 9. Energetics associated with intron toggling in the non-protonated state. a) Structural architecture of the active site for each state identified by the MtD simulation reported in panel b. b) Path metadynamics (MtD) free energy landscape of the cleaved state. The cleaved, A', B', and toggled states are indicated along the MtD trajectory (dark blue dotted line). The energy scale is indicated in kcal mol^{-1} on the right. The conformations of the J2/3 junction in state A' and in the toggled state are represented at the bottom of the figure.

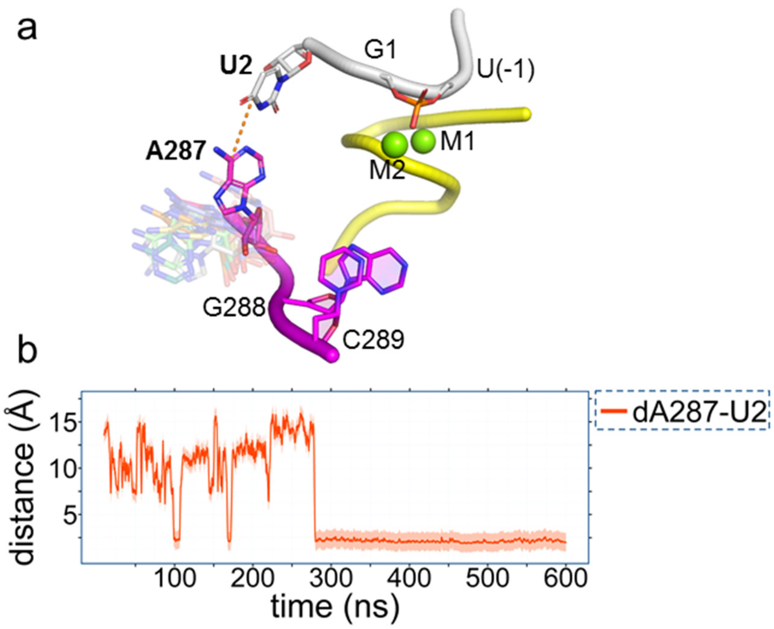

Supplementary Figure 10. A287-U2 interaction. a) Graphical representation of the stabilized base pair between A287 with U2 (red dotted line indicated $d_{U2-A287}$). b) Evolution of $d_{U2-A287}$ during the MD simulation of the pre-hydrolytic state. Shading around the traces indicates the standard deviation of the corresponding distance.

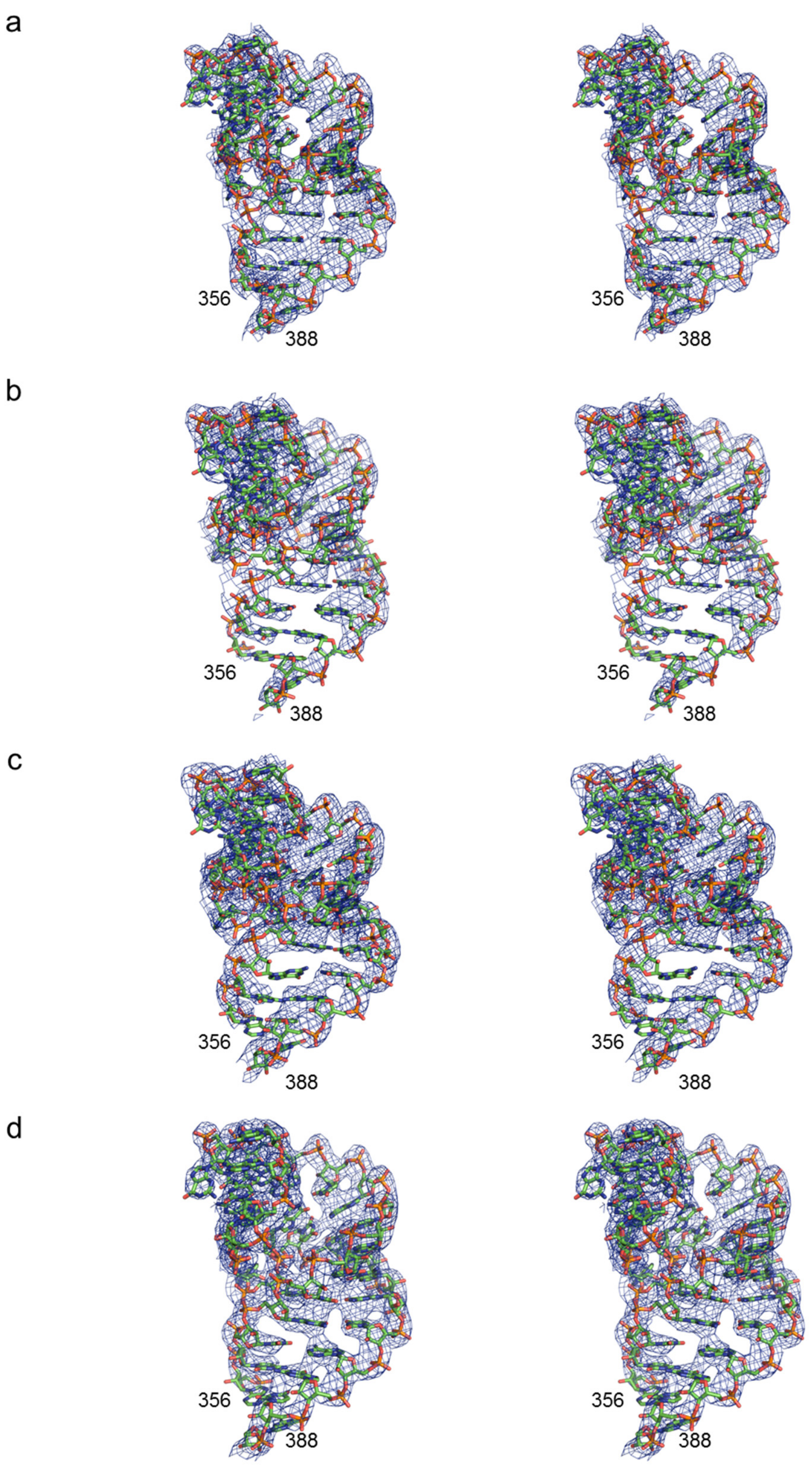

Supplementary Figure 11. Stereo images of the electron density maps. Wall-eye stereo images of $2F_o$-F_c electron density maps of D5 (nucleotides 356-388) for the structures presented in this work. a) G-mutant in potassium and magnesium (1.5 σ contour level). b) U-mutant in potassium and magnesium (1.5 σ contour level). c) G-mutant in potassium and sodium (1.0 σ contour level). d) U-mutant in potassium and sodium (1.5 σ contour level). The RNA is depicted as sticks, color coded by atom type (carbon atoms in green, oxygen in red, nitrogen in blue, and phosphorus in orange). The electron density is depicted as a blue mesh.

Supplementary Tables

Supplementary Table 1. pK_A values of adenine and cytosine residues in the group II intron active site, as empirically estimated using continuum electrostatics non-linear Poisson-Boltzmann calculations. We note that these calculations provide only qualitative approximations of the tendency of the reported nucleotides to shift pK_A in different conformational states.

Residue	Precatalytic state (PDB id.: 4FAQ)	Toggled state (PDB id.: 4FAX)
**J23 junction**		
A287	6.6	7.3
C289	7.0	8.5
**cat triad**		
C358	4.5	7.2
C360	3.2	3.7
**2nt-bulge**		
A376	11.9	9.7
C377	10.3	0.7

Supplementary Table 2. Kinetic rate constants of *O. iheyensis* wild type intron and A-, G-, and U-mutants. Standard errors of the mean were calculated from n = 3 independent experiments. The wild type and A-mutant show a double exponential decay of precursor, so the kinetic rate constants of both the fast and slow populations are reported for these two constructs. The relative sizes of each population are indicated in the right column. Source data are provided as a Source Data file.

Construct	k_1 (min^{-1})		k_2 (min^{-1})		population size	
WT	fast:	0.133 ± 0.014	fast:	0.096 ± 0.015	fast:	64 %
	slow:	0.007 ± 0.003	slow:	0.006 ± 0.004	slow:	36 %
A-mutant	fast:	0.075 ± 0.009	fast:	0.045 ± 0.008	fast:	73 %
	slow:	0.008 ± 0.006	slow:	< 0.006	slow:	27 %
G-mutant		0.011 ± 0.001		0.002 ± 0.001		
U-mutant		0.019 ± 0.001		0.012 ± 0.001		
