# Supplementary material for: Visualizing group II intron dynamics between the first and second steps of splicing
Source: Nat Commun. 2020 Jun 5;11:2837. doi: 10.1038/s41467-020-16741-4 (PMC7275048; doi:10.1038/s41467-020-16741-4)
Supplement: Supplementary file 3 — Reporting Summary [file 41467_2020_16741_MOESM3_ESM.pdf]

nature research

Corresponding author(s): Marco De Vivo, Marco Marcia

Last updated by author(s): Apr 30, 2020

Reporting Summary

Nature Research wishes to improve the reproducibility of the work that we publish. This form provides structure for consistency and transparency in reporting. For further information on Nature Research policies, see Authors & Referees and the Editorial Policy Checklist.

Statistics

For all statistical analyses, confirm that the following items are present in the figure legend, table legend, main text, or Methods section.

n/a	Confirmed	
☐	☒	The exact sample size (*n*) for each experimental group/condition, given as a discrete number and unit of measurement
☐	☒	A statement on whether measurements were taken from distinct samples or whether the same sample was measured repeatedly
☒	☐	The statistical test(s) used AND whether they are one- or two-sided<br>*Only common tests should be described solely by name; describe more complex techniques in the Methods section.*
☒	☐	A description of all covariates tested
☒	☐	A description of any assumptions or corrections, such as tests of normality and adjustment for multiple comparisons
☐	☒	A full description of the statistical parameters including central tendency (e.g. means) or other basic estimates (e.g. regression coefficient) AND variation (e.g. standard deviation) or associated estimates of uncertainty (e.g. confidence intervals)
☒	☐	For null hypothesis testing, the test statistic (e.g. *F*, *t*, *r*) with confidence intervals, effect sizes, degrees of freedom and *P* value noted<br>*Give P values as exact values whenever suitable.*
☒	☐	For Bayesian analysis, information on the choice of priors and Markov chain Monte Carlo settings
☒	☐	For hierarchical and complex designs, identification of the appropriate level for tests and full reporting of outcomes
☒	☐	Estimates of effect sizes (e.g. Cohen's *d*, Pearson's *r*), indicating how they were calculated

Our web collection on statistics for biologists contains articles on many of the points above.

Software and code

Policy information about availability of computer code

Data collection	Rapid Automated Processing of Data (RAPD; NE-CAT beamlines, Argonne Photon Source; https://rapd.nec.aps.anl.gov/ and https://cci.lbl.gov/dials/3/1040_jon.pdf); Gromacs molecular dynamics (MD) engine v 5.1.4 (Abraham, 2015); Plumed v 2.3 (Bonomi, 2009 and Branduardi, 2012); MolMovDB (Krebs, 2000)
Data analysis	GraphPad Prism v 6 (GraphPad Software); XDS v. March 30th 2013 (Kabsch, 1993); Phenix v 1.14-3260 (Adams, 2010); Refmac5 v 5.7.0032 (Murshudov, 1997); Coot v 0.8.9.2 (Emsley, 2004); MolProbity v 4.02b-467 (Chen, 2010); PyMOL v 1.8.0.4 (Schrodinger LLC); CCP4 v 7.0 (Collaborative Computational Programme number 4, 1994); P-LINCS (Hess, 2008).

For manuscripts utilizing custom algorithms or software that are central to the research but not yet described in published literature, software must be made available to editors/reviewers. We strongly encourage code deposition in a community repository (e.g. GitHub). See the Nature Research guidelines for submitting code & software for further information.

Data

Policy information about availability of data

All manuscripts must include a data availability statement. This statement should provide the following information, where applicable:

- Accession codes, unique identifiers, or web links for publicly available datasets
- A list of figures that have associated raw data
- A description of any restrictions on data availability

Coordinates and structure factors have been deposited in the Protein Data Bank under accession codes 6T3K, 6T3R, 6T3N, and 6T3S. Data supporting all other findings of this manuscript, including MD simulation trajectories, are available from the corresponding authors upon request. A reporting summary for this Article is available as a Supplementary Information file. The source data underlying Figure 1B/C and Supplementary Table S2 are provided as a Source Data file.

Field-specific reporting

Please select the one below that is the best fit for your research. If you are not sure, read the appropriate sections before making your selection.

☒ Life sciences ☐ Behavioural & social sciences ☐ Ecological, evolutionary & environmental sciences

For a reference copy of the document with all sections, see nature.com/documents/nr-reporting-summary-flat.pdf

Life sciences study design

All studies must disclose on these points even when the disclosure is negative.

Sample size	Sample size determination is not applicable to our study. All enzymatic assays were performed in triplicates, in agreement with previous literature in the field of splicing. All MD simulations were performed in 2-6 independent replicas counting a total of at least 1 microsecond simulation per system. This procedure allows for an extensive sampling of the conformational space. Enhanced sampling MD calculations were run till convergence was reached.
Data exclusions	No data were excluded from the analysis.
Replication	All kinetics could be reproduced successfully in at least three independent experiments. Experiments carried out even 1 year apart from each other yielded reproducible results. 2-6 MD simulations were performed under each condition, as indicated in the text and figures, and simulation results were also reliably reproducible by different authors.
Randomization	Randomization is not applicable to our study, because our study is based on structural models and is not conceived in the way other experiments are, where randomization is used as a control. The study design could not be randomized as analysis of simulations and experimental results required knowledge of the identity of each sample. Randomization does not apply to MD simulations as the identity of the samples are known a priori, since MD simulations are based on structural models.
Blinding	Blinding is not applicable to our study, because our study presents crystal structures and computational simulations of crystal structures. Thus, it does not require blinding as a control, unlike other type of experiments. There is no way to crystallize a molecule or to run MD simulations accounting for blinding. Blinding does not apply to crystallographic analysis, enzymatic assays, or MD simulations as the identity of the samples are known a priori.

Reporting for specific materials, systems and methods

We require information from authors about some types of materials, experimental systems and methods used in many studies. Here, indicate whether each material, system or method listed is relevant to your study. If you are not sure if a list item applies to your research, read the appropriate section before selecting a response.

Materials & experimental systems

n/a	Involved in the study
☒	☐ Antibodies
☒	☐ Eukaryotic cell lines
☒	☐ Palaeontology
☒	☐ Animals and other organisms
☒	☐ Human research participants
☒	☐ Clinical data

Methods

n/a	Involved in the study
☒	☐ ChIP-seq
☒	☐ Flow cytometry
☒	☐ MRI-based neuroimaging
