# Supplementary material for: Visualizing group II intron dynamics between the first and second steps of splicing
Source: Nat Commun. 2020 Jun 5;11:2837. doi: 10.1038/s41467-020-16741-4 (PMC7275048; doi:10.1038/s41467-020-16741-4)
Supplement: Supplementary file 4 — Description of Additional Supplementary Files [file 41467_2020_16741_MOESM4_ESM.pdf]

Description of Additional Supplementary Files

File Name: Supplementary Movie 1
Description: The movie is a morph between crystal structures and MD simulations described in this work. It depicts the intron active site evolving from the pre-hydrolytic state to the toggled state, including the attack of the nucleophilic water, cleavage of the scissile phosphate, protonation of C358, release of K1, and toggling of the J2/3 junction.
